# Measuring factors associated with identification thresholds in fingerprint analysts

**DOI:** 10.1111/1556-4029.70085

**Published:** 2025-05-19

**Authors:** Nada Aggadi, Meredith Coon, Thomas Busey

**Affiliations:** ^1^ Department of Psychological Brain and Sciences Indiana University Bloomington Indiana USA; ^2^ AVER, LLC Washington District of Columbia USA

**Keywords:** decision‐making, fingerprint examiners, general self‐efficacy, identification thresholds, need for closure, stress

## Abstract

The goal of this research is to measure and identify factors associated with identification thresholds in fingerprint analysts. Conclusions reached in friction ridge comparisons require the application of individual thresholds. While previous studies have investigated the mechanisms behind value determinations, or on the perceived stress reported by forensic examiners, only a few have focused on the influence of personality traits and environmental factors on identification thresholds. This study measures how individual traits and workplace policies may shape these thresholds. Participants were presented with latent prints and tasked with making value determinations before conducting a latent print comparison. Post‐trial, participants responded to a series of survey inquiries focusing on their personalities and interactions within the work environment. Our results demonstrate a significant positive correlation between Need for Closure score and the number of Identification decisions, as well as a significant positive correlation between Stress levels and the number of Identification decisions.

**Highlights**

- Fingerprint examiners develop their own personal, internal thresholds for sufficiency.
- Factors such as stress and personality traits can influence decision‐making.
- Participants with higher Need for Closure are more likely to make identifications.
- Participants with higher stress levels were more likely to make identifications.
- This study emphasizes the need for standardized communication practices.

## 1 INTRODUCTION

In forensic science, the potential for human error is a key concern, particularly in disciplines like fingerprint identification that rely on a series of subjective judgments throughout the decision‐making process. Indeed, experts must make discretionary decisions with limited standardized criteria, introducing opportunities for variability and subjectivity that can contribute to errors. Dror [1] discussed how forensic experts produce inconsistencies in their decision‐making, both across examiners (lack of reproducibility) and within an examiner across time (lack of repeatability). This contrasts with expectations of the judicial system, which assumes that forensic experts provide independent evidence that has been obtained in a scientifically rigorous manner [2]. The inconsistencies observed in many forensic disciplines highlight the need to study factors that contribute to this variability, and the current work addresses factors that affect decision‐making in fingerprint examinations.

Latent print examiners compare fingerprint impressions found at crimes to known prints from suspects to determine whether the impressions have come from the same source. To do so, experts assess the level of agreement between the two prints, looking for features that are common in their placement and appearance, but also any features that differ and cannot be attributed to the particular way that impression was recorded. In the US, fingerprint examiners follow the ACE‐V method (Analysis, Comparison, Evaluation, and Verification) to conduct fingerprint comparisons. During the analysis phase, the examiner assesses the quality and sufficiency of the latent print to determine whether it contains enough detail to proceed with a comparison [3]. The comparison phase involves evaluating the perceptual similarity between the latent print and an exemplar or database candidate. The examiner evaluates whether the prints originate from the same source (Identification/Inclusion), from different sources (Exclusion), or if there is insufficient evidence to reach a conclusion (Inconclusive). In the US, the way that examiners communicate the results of a fingerprint comparison can vary by jurisdiction and based on which agency or laboratory they work for as there are no national‐wide standards. Typically, though, examiners use a verbal categorical scale that varies in granularity. Some scales have just three categories—“Identification/Inclusion” (their analysis, comparison, and evaluation led them to believe they are from the same source), “Exclusion” (their analysis, comparison, and evaluation led them to believe they are from different sources), and Inconclusive (their analysis, comparison, and evaluation did not provide them with enough evidence to conclude either way). Even in the best of circumstances, forensic evidence is incomplete or ambiguous and requires interpretation by experts. Experts must make subjective judgments based on their experience, training, and environment, and their decisions can be influenced by a variety of factors, both internal and external [4, 5, 6].

OSAC and other organizations provide proposed standards, but they do not have regulatory powers. Since there are no clear guidelines from United States policymakers on what constitutes sufficient observations for a definitive conclusion such as Identification, fingerprint examiners typically rely on their experience with ground truth samples from their training to develop their own personal, internal thresholds for sufficiency. These thresholds are reinforced and refined through exposure to casework materials, interactions with peers through verification procedures, and proficiency tests. Examiners also get some perceptual feedback during the comparison and may eventually get some feedback from the other facts in the case. However, in the end, fingerprint examiners use their personal judgment to decide when they have enough evidence to reach an Identification. It would be naive to assume that every expert has the same decision thresholds for identification [7]. As a matter of fact, examiners can vary widely in their identification thresholds, even when presented with the same evidence.

This variability is due to a number of factors. Some, such as education level, experience, and training, have not been found to be correlated with identification thresholds [8]. Researchers have identified intrinsic factors such as cognitive biases [9], as well as extrinsic factors such as the size of the AFIS database [10], laboratory requirements to reach Identification [11], or the type of crime investigated [12]. Indeed, examiners might alter sufficiency or ID threshold based on the crime type, with high violent crime increasing the likelihood of reaching a conclusion. Finally, Langenburg, Champod, and Wertheim [13] highlighted the influence of colleagues on identification threshold, showing that experts were influenced by contextual information during fingerprint comparisons. The goal of our research is to identify and measure some of the factors that may contribute to this variability.

In the current study, we measured each examiner's Identification threshold in a task using comparisons of borderline images with a traditional three‐conclusion scale. We chose image pairs that, in previous studies, received a mixture of Support for Common Source and Identification responses and therefore were likely to exhibit variation across examiners in the present work. This variation could then be associated with individual factors, as described next.

## 2 STRESS

Examiners experience stress for a variety of reasons [14], including laboratory backlogs, the violent nature of crime, and the lack of clear guidance on identification thresholds [15]. The high workload is one of the main factors contributing to stress in fingerprint analysts. Examiners are often under pressure to process cases quickly and efficiently, even when they are dealing with complex cases or a large number of cases. Most importantly, they are expected to not make any mistakes in the process [9]. This can lead to examiners feeling overwhelmed and burned out [16]. Some examiners are exposed to graphic and disturbing crime scene evidence, which can take a toll on their mental health. In a study by Hall [6], fingerprint experts were examined to determine whether an emotional context would influence their judgment of an ambiguous or low‐quality fingerprint. The findings suggested that the emotional context had a perceived impact, as more experts reported being affected by the information they received. Additionally, the pressure to make accurate identifications is high, as mistakes can have serious consequences for their careers and for society at large [17]. Examiners feel that stress originates mainly from managers and supervisors [16]. The literature covering the impact of stress on decision‐making is mixed and complex. Indeed, some studies have proposed an association between stress and risk‐taking in fingerprint examiners [18]. Studies in the general population have concluded that stress increases risk‐taking behavior [19].

Examiners are subject to several sources of daily stress, and this paper is an attempt to measure their levels of stress and determine its possible effects on the identification threshold. Chronic stress can be measured using the 9‐item Trier Inventory for Chronic Stress (9‐TICS) [20]. 9‐TICS is a standardized questionnaire for assessing nine interrelated factors of chronic stress derived from the original TICS, such as pressure to perform, work discontent, or excessive demands at work (TICS‐57) [21].

## 3 PERSONALITY

Personality is a broad concept that combines a variety of individual traits, including temperament, character, and behavior [22]. Personality can have a significant impact on decision‐making [23]. For example, people with low levels of conscientiousness are more likely to make deliberate and thoughtful decisions, while people with low levels of openness are more likely to make impulsive and emotional decisions [24]. These same personality traits may influence decision‐making in fingerprint analysts while performing their work. This research focuses on Need for Closure (NFC) and General Self‐Efficacy (GSE).

Need for Closure is the desire for certainty and finality, and it can have a significant impact on how people make decisions in a variety of contexts [25]. In the literature, high Need for Closure is associated with two tendencies: The tendency to reach closure as soon as possible is referred to as “seizing,” and the tendency to maintain closure for as long as possible is referred to as “freezing” [26]. Studies have shown that people with high Need for Closure were more likely to make judgments about ambiguous stimuli, even when they were instructed to avoid doing so [26]. Additionally, people with high Need for Closure were more likely to accept premature closure on complex problems, even when they had access to additional information [26, 27]. High Need for Closure has also been shown to predict higher stress levels [28, 29], a factor that is already characteristic of the forensic environment, as we explained earlier. Need for Closure has been studied in fingerprint analysts during a study by Charlton, Fraser‐Mackenzie [30] investigating the emotional and motivational factors involved in fingerprint analysis. By conducting a series of interviews, results showed that examiners expressed a strong desire for closure, wanted to avoid ambiguity, saw cases through to completion, and accounted for all evidence in order to find a definitive solution. This study found evidence for Need for Closure in fingerprint examiner decision‐making, especially when it related to high‐profile, long‐running, or serious crimes. For those reasons, we hypothesized that experts with high Need for Closure would make more Identifications.

General Self‐Efficacy (GSE) is a personality trait that reflects a person's belief in their ability to cope with challenges and succeed in difficult situations [31]. There is a strong link between general self‐efficacy (GSE) and stress. People with high GSE tend to experience lower levels of stress in response to stressful events [32, 33, 34, 35]. Because of this, we consider that GSE is particularly relevant to fingerprint analysts, who are required to make difficult decisions under pressure. We anticipate that experts with high GSE will have higher identification thresholds, as we believe that experts with high GSE are more cautious in their decision‐making, which in turn results in higher identification thresholds. Their self‐regulatory tendencies may lead them to commit only when they perceive the evidence as sufficiently clear, thereby producing high confidence judgments. In this view, confidence is not the driver of caution, but rather a consequence of their deliberate approach—decisions are made only when they are certain enough to feel confident.

So far, the field has not measured the relation between identification thresholds and individual factors. The goal of this study is to measure the strength of the association between the identification threshold, stress, and personality traits (Need for Closure and GSE). To do so, participants were presented with latent prints and conducted pairwise comparisons with two prints. We included 14 traditional comparisons, as well as two sequential reveal tasks that have the potential to be more sensitive to the exact individual threshold for each examiner. After making these comparisons, participants responded to the 9‐TICS, Need for Closure, and GSE questionnaires.

We hypothesize that examiners with higher Need for Closure and Stress levels will exhibit lower identification thresholds, making more Identification decisions due to a greater drive for decisiveness, while those with higher General Self‐Efficacy will have higher identification thresholds, demonstrating greater caution in committing to an Identification. Details of the specific methods are provided below, but a brief summary of the fingerprint comparison tasks follows. The fingerprint comparison phase of the experiment contained three parts, and all participants proceeded through all comparisons in the same order with no opportunity to skip an image pair or move to the next phase without completing the current comparison. The first part of the comparison phase involved 14 traditional casework‐like comparisons. After completing all 14 comparisons, the participant completed two sequential reveal tasks, in which a small portion of the latent print was presented for comparisons. Once a decision was made, a small portion of the latent print was added to the latent print and the participant made an additional comparison decision using the traditional three‐conclusion scale. Each sequential reveal task had 13 image reveals. The final estimate of a participant's Identification threshold was calculated as the total number of identification decisions across both the 14 traditional comparisons and the two sequential reveal sequences. Additionally, we assessed each participant's stress levels and personality traits using validated psychological measures. By integrating these factors, our study seeks to clarify the role of cognitive and emotional influences in forensic decision‐making.

## 4 METHOD

### 4.1 Participants

We recruited 77 active latent print examiners for our study. Of these, 7 began the first part of the experiment but did not complete it, and 6 withdrew for personal reasons. Ultimately, we collected data from 56 latent print examiners. Subjects were recruited via email and were not compensated for their participation in the study. All participants were at least 18 years old, had at least a year of active latent print casework, and self‐reported normal or corrected‐to‐normal vision. All participants were from different labs within the United States. We chose not to record participants age, gender, certification status, laboratory accreditation status, or years of experience. Prior studies have not found associations between these variables and identification thresholds [32], and measuring these associations would lead to alpha inflation—the heightened risk of false positives that arises when multiple statistical comparisons are conducted without correction—leading to an increased possibility of spurious correlations.

### 4.2 Design and material

We used ground truth image pairs from the Busey, Klutzke, Nuzzi, and Vanderkolk [15] study. The Busey et al. study relied on a Black Box design to evaluate scientific validity in comparison disciplines [2]. In those studies, researchers treat the examiner as a “black box” in order to measure the accuracy of the system without detailed knowledge of the underlying processes.

For our study, we selected 14 mated pairs from the Busey et al. [15] dataset, specifically targeting pairs that had a range of responses from examiners including both “Inconclusive” and “Identification” decisions. Selecting such borderline mated pairs provides an efficient way to estimate the threshold between Inconclusive and Identification. We discuss this choice and its implications further in the Discussion section.

**FIGURE 1:**
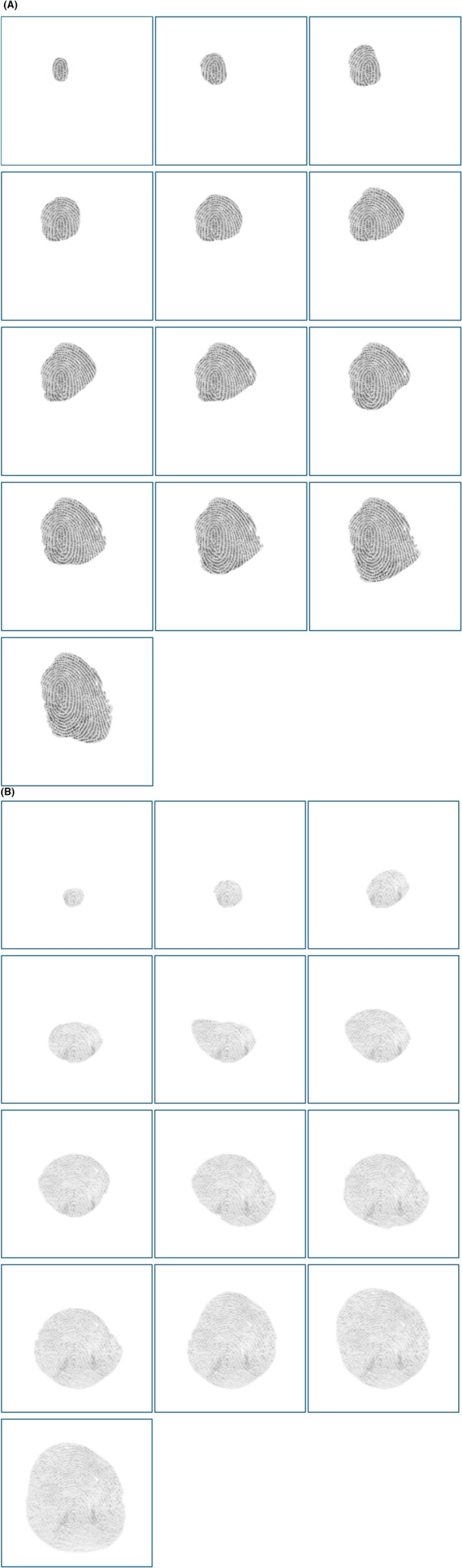
(A) Sequential reveals of the latent print from Sequence 1. Participants made an “Of Value” and conclusion decision on each fragment prior to being presented with the next fragment, which is a proper superset of the prior fragment. See the OSF site for the full‐resolution images used in the study. (B) Sequential reveals of the latent print from Sequence 2. Participants made an “Of Value” and conclusion decision on each fragment prior to being presented with the next fragment, which is a proper superset of the prior fragment. See the OSF site for the full‐resolution images used in the study.

In addition to these 14 comparisons, we included two additional image pairs in the form of a sequential reveal process. For each of the two image sequences, examiners initially saw only a small portion of the latent print and made a decision using the Exclusion/Inconclusive/Identification scale. After this, an additional region was revealed on the latent print, requiring another “Of Value” and comparison decision. This process was repeated 13 times in each sequence. Figure 1 illustrates the 13 stages of both sequential reveals.

Examiners performed all comparisons under the following definitions:

*Exclusion*: Exclusion is the conclusion that two friction ridge impressions did not originate from the same source. Exclusion is reached when in the examiner's opinion, considering the observed data, the probability that the two impressions came from the same source is considered negligible.

*Inconclusive*: The observed characteristics of the items are insufficient to support any of the other conclusions (including one of the “support” conclusions if they are available).

*Identification*: Identification is the strongest degree of association between two friction ridge impressions. It is the conclusion that the observations provide extremely strong support for the proposition that the impressions originated from the same source and extremely weak support for the proposition that the impressions originated from different sources. Identification is reached when the friction ridge impressions have corresponding ridge detail and the examiner would not expect to see the same arrangement of details repeated in an impression that came from a different source.

Examiners were also given conditioning information: “Once you finish your value determination, we will show you an exemplar print which was the result of the first candidate of a regional AFIS database search containing 1 million records. We would like you to conduct a latent print comparison. We would like you to use the following definitions when making your conclusions.” Figures illustrating the interface and instructions provided to examiners are available in the Figures S1 and S2.

### 4.3 Measuring identification thresholds

The study was reviewed and approved by the Indiana University Institutional Review Board (IRB), and all participants were enrolled with an anonymous user id and password. Participants logged into the site and read and electronically consented using an online consent form.

For the first 14 trials, examiners were presented with a latent print and asked to make a value determination using the following options: No value, Of Value for Exclusion, Of Value for Exclusion or Identification. Regardless of their decision, examiners then performed a comparison where they were presented with an exemplar print and asked to reach a definitive conclusion using a three‐conclusion scale (Identification/Inconclusive/Exclusion). Examiners were able to interact with the prints, including adding colored dots or lines to the impressions, zooming, and rotating images.

To provide a potentially more sensitive measure of an examiner's identification threshold, examiners performed two additional comparisons in which portions of the latent were sequentially revealed. We started each of the two sequences by showing examiners only a small portion of the latent print and asked them to make a value decision (Figure 1A,B). Then, we presented an exemplar alongside the latent print and asked them to make a comparison using a three‐conclusion scale: Identification, Inconclusive, Exclusion. After making a response, a small additional region of the latent was revealed such that each trial gradually revealed more of the latent print as shown in Figure 1A,B. After each portion was revealed, we repeated both the suitability and conclusion decisions at each step. Each sequential reveal started with a very small region and ended with the full latent print revealed. As with previous trials, examiners were able to interact with the prints, including marking up prints, zooming, and rotating images. The sequential reveal part of the experiment was done twice with two different latent and known pairs. We designed each sequence such that the first region was unlikely to produce an Identification conclusion, and when the entire latent was revealed, most examiners would reach an Identification conclusion. Thus, the step at which an examiner first said Identification would represent the amount of detail in agreement they believed would justify saying Identification, and we believed that this would be a very sensitive measure of an examiner's personal Identification threshold while still maintaining most aspects of a traditional latent print comparison. As we will see, Sequence 1 was of higher quality and had limited success at this goal because it produced a restricted range of responses. However, Sequence 2 had greater variability across examiners.

### 4.4 Measuring our factors of interest

After they completed the 40 comparisons, they were directed to a Qualtrics survey where answered the following statements: “I find my laboratories procedures towards false positives to be overly punitive,” “I am afraid mistakes could affect my professional reputation,” and “I am scared of losing my job if I make mistakes.” Subjects answered using the following scale: “Never,” “Rarely,” “Sometimes,” “Frequently,” and “Always.” Note that these statements are framed negatively, which may have primed negative answers. In the extreme, this may have restricted the range of responses and therefore reduced the size of the observed correlations.

The Need for Closure was measured with the Need for Cognitive Closure Scale [25]. The scale consists of 41 items divided into five facet scales: need for order, need for predictability, decisiveness, avoidance of ambiguity, and closed‐mindedness. Example items: I don't like situations that are uncertain or I don't like to be with people who are capable of unexpected actions. The answers are given on a 6‐point Likert scale from definitely disagree to definitely agree. Higher scores indicate higher Need for Closure. (Cronbach's *α* = 0.80, *M* = 3.53, SD = 0.66, ranging from 1.58 to 5.25).

The General Self‐Efficacy Scale (GSE) measures one's level of determination and resilience in pursuing endeavors despite facing physical or emotional challenges [31]. Participants rate items on a 7‐point Likert scale ranging from 1 for “Completely Disagree” to 7 for “Completely Agree.” Example statements include “I am confident that I could deal efficiently with unexpected events.” and “I can always manage to solve difficult problems if I try hard enough.” High GSE facilitates goal‐setting, effort investment, persistence in face of barriers, and recovery from setbacks. GSE has been found to be a very robust construct across cultures. Data from 23 countries showed that Cronbach's alphas ranged from 0.76 to 0.90, with the majority in the high 0.80s.

To measure participants' chronic stress levels, we used the 9‐item version of the Trier Inventory for Chronic Stress (TICS‐9) [20]. Each item was rated in a self‐assessment on a five‐point Likert scale in respect to how often the test subjects had experienced a certain situation or made certain experiences within the last 3 months (0 = never, 1 = rarely, 2 = sometimes, 3 = often, 4 = very often). Each question in TICS‐9 is designed to evaluate nine chronic stressors: Work Overload, Social Overload, Pressure to Perform, Work Discontent, Excessive Demands at Work, Lack of Social Recognition, Social Tensions, Social Isolation, and Chronic Worrying.

In the instructions, we did not emphasize reaction time, and predictions from the various theoretical accounts are ambiguous with respect to associations with reaction time. The interface contained a 30‐min countdown timer to make the task tractable, and a pause button temporarily hid the images if the examiner was interrupted during the tasks. However, an examiner could have been momentarily distracted, which makes response time a difficult measure of time on tasks. Because of these complications, we have chosen not to analyze response times.

### 4.5 Data analysis

We estimated a subject's identification threshold as the number of prints they identified. To do so, we created a summary score called “Total Number of IDs” from the number of Identifications made in the first 14 comparisons and in the two sequential reveals in the experiment. Note that because the first phase had 14 traditional comparisons and each of the two reveal sequences have 13 steps, this summary score is approximately equivalent to computing the percentage of Identification decisions in each of the three phases and averaging to compute a single summary score of percent Identification decisions across all trials. We measured the association between the Total Number of IDs, Need for Closure, GSE, Stress Levels, and laboratory procedures. We also computed the correlations with the three individual phases and the factors.

## 5 RESULTS

For all analyses, we adopt an alpha level threshold of 0.05 and did not correct for multiple tests. We performed frequentist correlations in the JASP software package [36].

**FIGURE 2:**
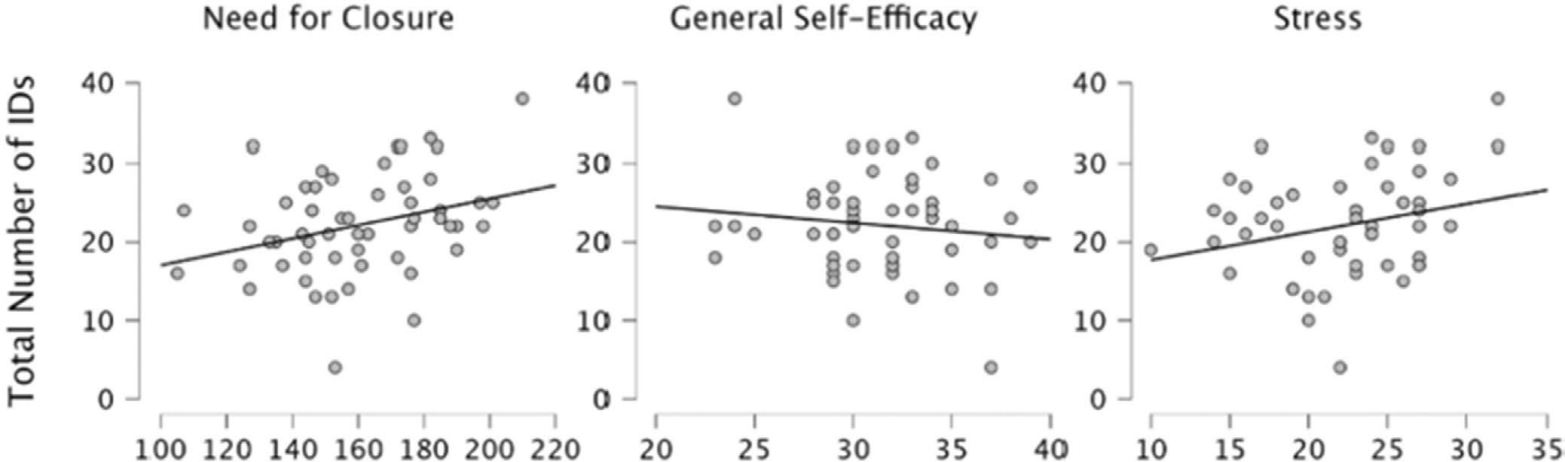
Correlation between Total Number of IDs and NFC, GSE, and Stress.

For Need for Closure, the mean score was 159.82 (SD = 23.22), with a minimum value of 105 and a maximum of 210. GSE had a mean score of 31.52 (SD = 3.76), with a minimum of 23 and a maximum of 39. Stress showed a mean of 22.32 (SD = 4.71), with a range from 10 to 30. The results of the analyses are summarized in Table 1, Figures 2. There were significant correlations between several of the variables of interest. As predicted, a positive correlation was found between the Total Number of IDs and Need for Closure (*r* = 0.313, *p* = 0.019), indicating that participants with higher Need for Closure tended to identify more prints overall. Stress levels also showed a significant positive correlation with the Total Number of IDs (*r* = 0.267, *p* = 0.047), illustrating an association between increased stress and a greater number of identifications, although we did not have a clear a priori prediction for the direction of this association.

**TABLE 1:** Correlation scores between Need for Closure, GSE, Stress, and the Total Number of IDs.

Pearson's correlations				
Variable	Total number of IDs	Need for closure	General self‐efficacy	Stress
1. Total Number of IDs				
Pearson's *r*	–			
*p*‐value	–			
2. Need for Closure				
Pearson's *r*	0.313	–		
*p*‐value	0.019	–		
3. General self‐efficacy				
Pearson's *r*	−0.125	−0.344	–	
*p*‐value	0.359	0.009	–	
4. Stress				
Pearson's *r*	0.267	0.348	−0.386	–
*p*‐value	0.047	0.009	0.003	–

Need for Closure was positively correlated with Stress (*r* = 0.348, *p* = 0.009), indicating that participants with a higher Need for Closure reported greater stress levels. A significant negative correlation was observed between General Self‐Efficacy and Stress (*r* = −0.386, *p* = 0.003), suggesting that individuals with higher GSE experienced lower levels of stress. GSE was also negatively correlated with Need for Closure (*r* = −0.344, *p* = 0.009), indicating that those with a higher Need for Closure tended to have lower GSE. Finally, the correlation between laboratory procedures and other variables was generally non‐significant, with no notable relationships observed between feelings about laboratory procedures and any other factor.

**FIGURE 3:**
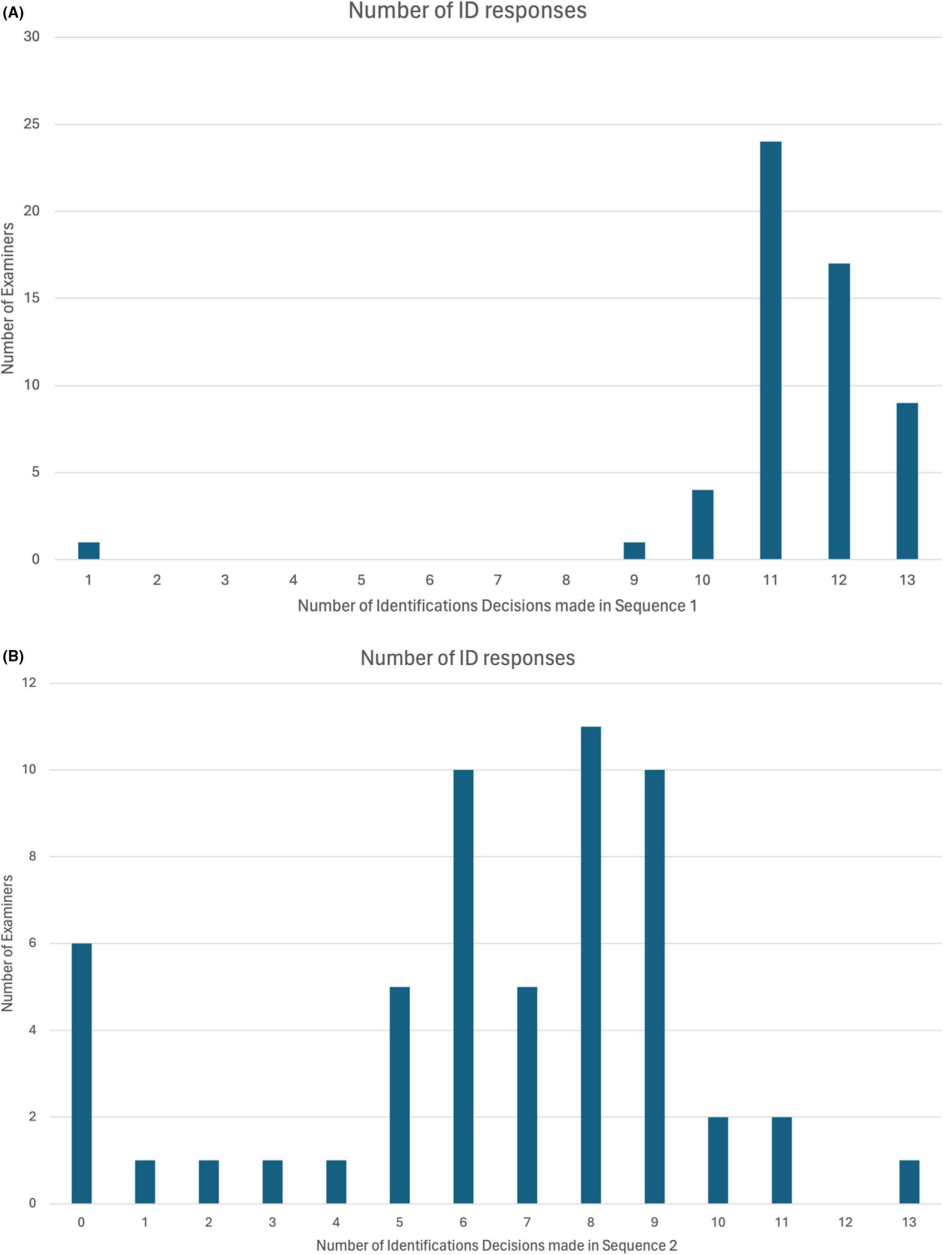
(A) Response distribution of Total Number of Identification for Sequential reveals in Sequence 1. Virtually all examiners made a large number of Identification decisions, implying that the image quality for even the smallest fragments was high enough to justify an Identification. Higher values imply examiners with lower identification thresholds (i.e., were willing to say Identification based on less information). (B) Response distribution of Total Number of Identification for Sequential reveals in Sequence 2. For example, six examiners never said Identification to any fragment in Sequence 2, while one examiner said Identification to all 13 fragments. Higher values imply examiners with lower identification thresholds (i.e., were willing to say Identification based on less information).

We also looked at the correlation between our factors of interest in our three different types of trials: Traditional Comparisons, which corresponds to the traditional comparisons, IDSeq1, which corresponds to the first sequential comparison, and IDSeq2, which corresponds to the second sequential comparison. As shown in Table 2, Figures 3, Need for Closure was positively correlated with the Traditional Comparisons (*r* = 0.232, *p* = 0.086, trend level significance) and IDSeq2 (*r* = 0.333, *p* = 0.012), suggesting that individuals with a high Need for Closure were more likely to make identifications in these trial sequences. However, IDSeq1 did not provide evidence for a significant association, which may be due to the restricted range that resulted from the high image quality of the latent. Additionally, a significant positive correlation was found between Traditional Comparisons and IDSeq1 (*r* = 0.321, *p* = 0.014), ID. Traditional Comparisons and IDSeq2 (*r* = 0.445, *p* < 0.001), as well as between IDSeq1 and IDSeq2 (*r* = 0.449, *p* < 0.001). These results demonstrate that all three sequences measure the same underlying threshold. Moreover, IDSeq2 had a slight negative correlation with GSE (*r* = −0.176, *p* = 0.195), though this was not statistically significant.

**TABLE 2:** Correlation scores between Need for Closure, GSE, Stress, and the Number of Identifications made during the Traditional Comparisons, Sequence 1 and Sequence 2.

Pearson's correlations					
Variable	Need for closure	GSE	Stress	Traditional	Seq1
1. NFC					
Pearson's *r*	–				
*p*‐value	–				
2. GSE					
Pearson's *r*	−0.344	–			
*p*‐value	0.009	–			
3. Stress					
Pearson's *r*	0.348	‐0.386	–		
*p*‐value	0.009	0.003	–		
4. Traditional					
Pearson's *r*	0.232	0.036	0.243	–	
*p*‐value	0.086	0.791	0.072	–	
5. Seq1					
Pearson's *r*	0.118	−0.210	0.173	0.327	–
*p*‐value	0.385	0.12	0.203	0.014	–
6. Seq2					
Pearson's *r*	0.333	−0.176	0.199	0.445	0.449
*p*‐value	0.012	0.195	0.141	<0.001	<0.001

Finally, we were interested in looking at the influence of Need for Closure, GSE, and Stress on Of Value decisions. To investigate this question, we summed up the number of times examiners said “Of Value for Exclusion” and “Of Value for Exclusion and Identification” into the variable Total Number of Sufficient. We did not find a significant associations between Need for Closure, GSE, Stress, and Total Number of Sufficient for any of our measures. This suggests that none of these factors has a moderate or strong association with our measured factors.

## 6 DISCUSSION

Our results indicate a significant positive correlation between the Need for Closure and identification thresholds, supporting our initial hypothesis that experts with higher Need for Closure would make more Identifications. The positive correlation between Need for Closure and the Total Number of Identifications suggests that participants with higher Need for Closure are more likely to make identifications, which aligns with the theoretical framework of Need for Closure. Individuals with high Need for Closure exhibit a strong desire for certainty and finality, often seeking to resolve ambiguity as quickly as possible. Indeed, inconclusive decisions do not provide any useful information on the case, leaving open the possibility of multiple interpretations [37]. As individuals with high Need for Closure are motivated to reduce uncertainty, inconclusive decisions become particularly problematic in situations where clarity and certainty are paramount, such as in legal or forensic contexts where decisions must be final and contribute to the overall case resolution. Therefore, inconclusive decisions fail to meet the cognitive and procedural demands of those seeking closure, and instead of aiding the decision‐making process, they perpetuate ambiguity.

The work environment may intensify high Need for Closure's desire to reach a conclusion, leading to a greater number of identifications as they work to eliminate ambiguity. We observed a positive correlation between stress levels and identification thresholds which aligned with our initial expectation that there would be an association. Participants with higher stress levels were more likely to make Identifications when presented with those ambiguous mated pairs. This behavior suggests that stress makes examiners more risk‐taking as their identification threshold is lower. The literature on the impact of stress on risk‐taking behavior is mixed. However, we can interpret those results as stress can heighten arousal levels. This phenomenon can result in a cognitive bias where examiners inadvertently lower their standards for making identifications. Research has shown that acute stress is enough to impair cognitive flexibility, simplifying strategies to make decisions under pressure [38]. Therefore, in the context of fingerprint analysis, stress may encourage examiners to conclude identifications more rapidly, even when the evidence is not entirely conclusive.

Our results showed a negative association between GSE and identification thresholds, though they were not significant. While the correlation did not reach statistical significance, the trend implies that as self‐efficacy increases, examiners might be more deliberate in their decision‐making processes, taking the time needed to ensure that their conclusions are well‐founded. This conclusion is supported by the fact that the strongest negative correlations were found between GSE, IDSeq1, and IDSeq2. Indeed, examiners with high GSE would eventually reach the correct conclusion that is Identification but require more of the latent print to be revealed before doing so. This suggests that high GSE could influence how latent print examiners approach uncertainty in decision‐making. Examiners with high GSE may feel more confident in their ability to make accurate judgments based on the available evidence, which could manifest in two distinct ways. First, they may not feel the need to seek additional information or external validation to support their conclusions, as they trust their own expertise and experience. This could lead to more independent decision‐making but might also increase the risk of overconfidence bias, where an examiner relies solely on their judgment without considering alternative interpretations or additional scrutiny.

Our method used only mated pairs and measured the number of Identification decisions. However, we expect a similar relationship between Need for Closure and exclusion decisions, with examiners high in Need for Closure likely to have a lower threshold for exclusion—meaning they may be more inclined to exclude prints rather than leaving the decision as inconclusive. Exclusion, like identification, provides a definitive resolution to a case, which aligns with the tendency of high Need for Closure individuals to seek closure and avoid ambiguity. This effect may be even more pronounced due to the procedural differences in fingerprint examinations: Exclusions are typically much faster decisions, often taking less than a minute, whereas identifications and inconclusive decisions require significantly more time due to the need for detailed documentation and justification. However, it is also important to consider agency‐specific protocols for exclusions, as these vary across laboratories and can influence how Need for Closure manifests in decision‐making.

While we anticipate a relationship between Need for Closure and exclusion decisions, we are less certain about how stress and GSE might influence exclusion thresholds because of the fewer consequences of erroneous exclusions. We are leaving potential relationships between Need for Closure, stress, GSE, and exclusion decisions as open questions for future research.

An examiner might reach an Identification decision because they are good (in the sense of a high signal detection d’ value), cocky (in the sense of a low decision threshold or requiring relatively little evidence before making an Identification decision), or both. To distinguish between these candidate mechanisms, we would have had to include nonmated pairs and used a large number of trials to measure erroneous identification outcomes. Erroneous identification outcomes are (fortunately) extremely rare (0.1% from [39]) making this difficult to measure. Thus, we cannot distinguish between ability and definitiveness, although such a distinction is also not possible in casework where ground truth is not known. Despite this, our conclusions have ecological validity even if we do not know whether our factors are associated more with expertise or definitiveness. This research does not address the influences of these traits on nonmated pair decisions, or the exclusion threshold as these are less consequential to the justice system. Future research on exclusion thresholds might distinguish between expertise and definitiveness as it relates to Need for Closure, GSE, and Stress. Additionally, the laboratories SOPs and verification and safeguards may also play a protective role in casework but were not tested in the present design.

Our goal with the two sequential reveal sequences was to provide a more sensitive measure of each examiners' identification threshold. We combined these with the results of the first 14 comparisons to compute an overall summary score that reflected our estimate of an examiner's identification threshold. We found significant and positive correlations between Traditional Comparisons, IDSeq1, and IDSeq2 indicating that the identifications made in each of these trials are highly related and likely assess the same process. This reinforces the idea that, despite the trials being distinct in their structure (traditional vs. sequential), they are all effectively measuring the same aspect of decision‐making: the examiner's threshold for making identifications. Note that the correlation between IDSeq1 and Need for Closure did not reach statistical significance, which may be a consequence of the restricted range of outcomes for this variable, as show in Figure 1A. The image quality was high enough that most examiners were willing to reach an identification conclusion based on only one, two, or three regions revealed. The reader is invited to inspect the images in Figure 1A to determine whether they agree that such small image detail is sufficient for the term Identification, but regardless, this sequence tended to provide only limited success at differentiating examiners in terms of their identification thresholds. Sequence 2 had much more variability and consequently a much higher correlation with Need for Closure and GSE.

A limitation of our study is that we did not explicitly ask participants to use the same thresholds from casework, and they may use a different definition in casework. We do not know if their responses might differ from casework; however, we tried to make the interface mimic a latent print examination as closely as possible, including many of the tools they may use. Thus, we view the task as at least approximately representative of casework.

### 6.1 Implications for casework

The present work informs several ongoing debates in the fingerprint community. The idea that examiners' identification thresholds depend on internal and environmental factors highlights a critical issue: The current methods by which examiners communicate their results rely on subjective interpretation. This subjectivity can lead to inconsistencies between examiners in how evidence is interpreted, potentially compromising the reliability of forensic conclusions. By acknowledging that factors such as stress and personality traits can influence decision‐making, we emphasize the need for standardized communication practices that minimize ambiguity and promote objectivity. Various organizations have suggested alternative articulation terms (or redefining existing ones) without sound scientific support for the terms or acknowledging the problem of using definitive conclusions [3, 40]. Pattern evidence disciplines have moved toward the inclusion of probabilistic reporting; however, this approach suffers from examiners hostility as well as a lack of understanding of the method [41]. Additionally, there has been discussions of how expanded conclusion scales or redefined conclusion scales might help mitigate some of these inconsistencies. Expanding conclusion scales or redefining them to include more granular categories could indeed create the risk that some latent print examiners may overrely on intermediate categories, such as “support for same source” or “strong support for same source.” However, individual difference factors could still influence how examiners use a more detailed scale.

A recently published paper [42] has demonstrated that a five‐response category scale provides clear advantages over two‐ and three‐response category scales across various metrics. Specifically, a five‐category scale produces a higher overall information gain, maintains high information gain even for risk‐averse examiners, is more tolerant of non‐optimal decision thresholds, and tends to encourage examiner decision thresholds that are closer to optimal. For an examiner who appropriately maps the available fingerprint evidence to the response scale, performance continues to improve as the number of response categories increases beyond five.

While no scale can completely eliminate individual differences in decision‐making, offering more granular options reduces the pressure to force a binary or three‐way decision and allows examiners to more precisely express their conclusions.

This paper's goal is not to suggest that some personality traits are better than others; instead, we want to encourage fact finders to acknowledge the influence of personality on decision thresholds. One way to counteract the influence of personality would be to rely on quantitative or probabilistic approaches such as likelihood ratios [43]; however, subjective and quantitative likelihood ratios might be subject to the influence of personality as well.

## CONFLICT OF INTEREST STATEMENT

The authors have no competing interests to declare.

## Supporting information


Figure S1.

Figure S2.

## Data Availability

The analyses generated for this study are available on Open Science Framework on the following link: https://osf.io/c2q3y/?view_only=db5779c84e994ea48e191cc7550d8404.
