# Supplementary material for: Measuring factors associated with identification thresholds in fingerprint analysts
Source: J Forensic Sci. 2025 May 19;70(5):1853–65. doi: 10.1111/1556-4029.70085 (PMC12424117; doi:10.1111/1556-4029.70085)
Supplement: Supplementary file 1 — Figure S1. [file JFO-70-1853-s002.docx]

FIG S1: Instructions provided to Examiners before they complete the 14 traditional comparisons and 2 sequential comparisons.
