# Supplementary material for: Measuring factors associated with identification thresholds in fingerprint analysts
Source: J Forensic Sci. 2025 May 19;70(5):1853–65. doi: 10.1111/1556-4029.70085 (PMC12424117; doi:10.1111/1556-4029.70085)
Supplement: Supplementary file 2 — Figure S2. [file JFO-70-1853-s001.docx]

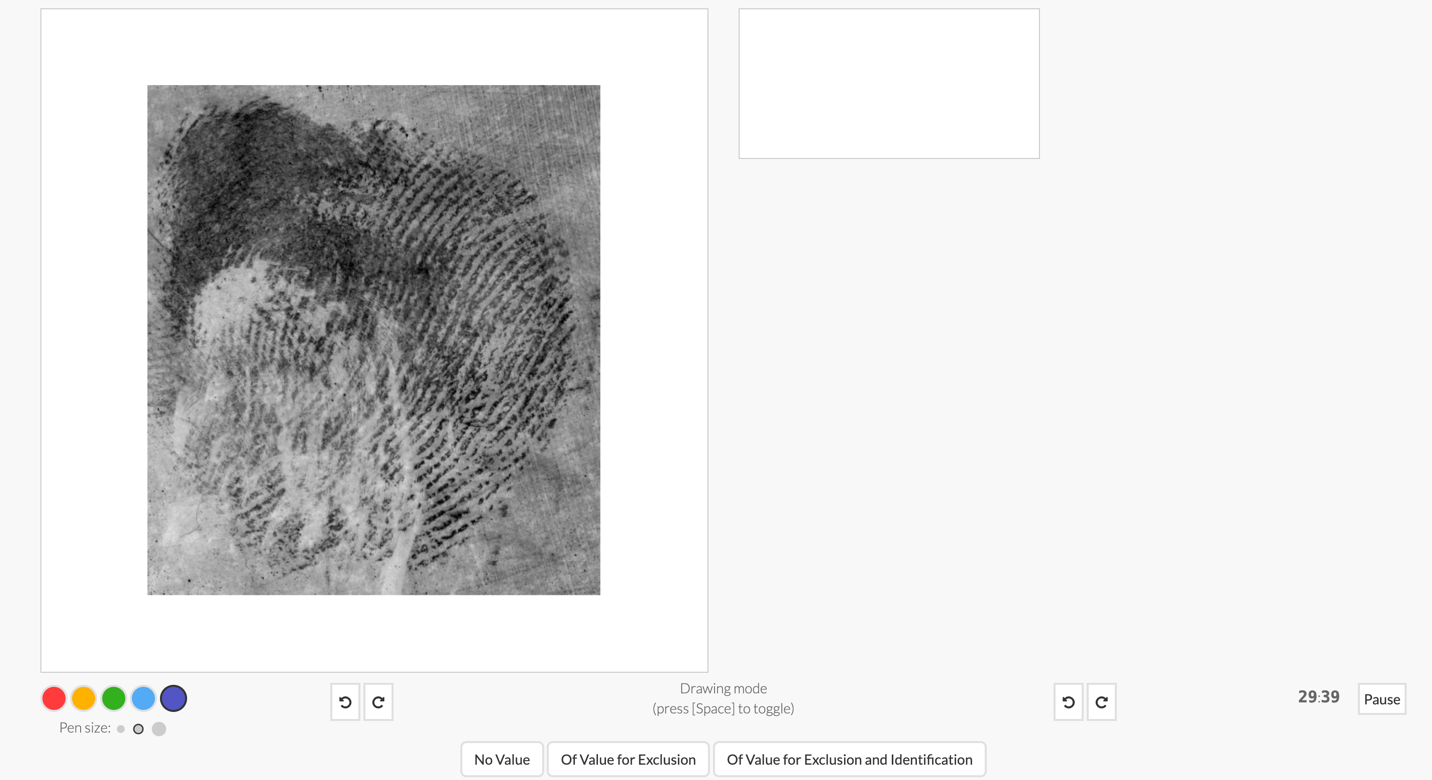


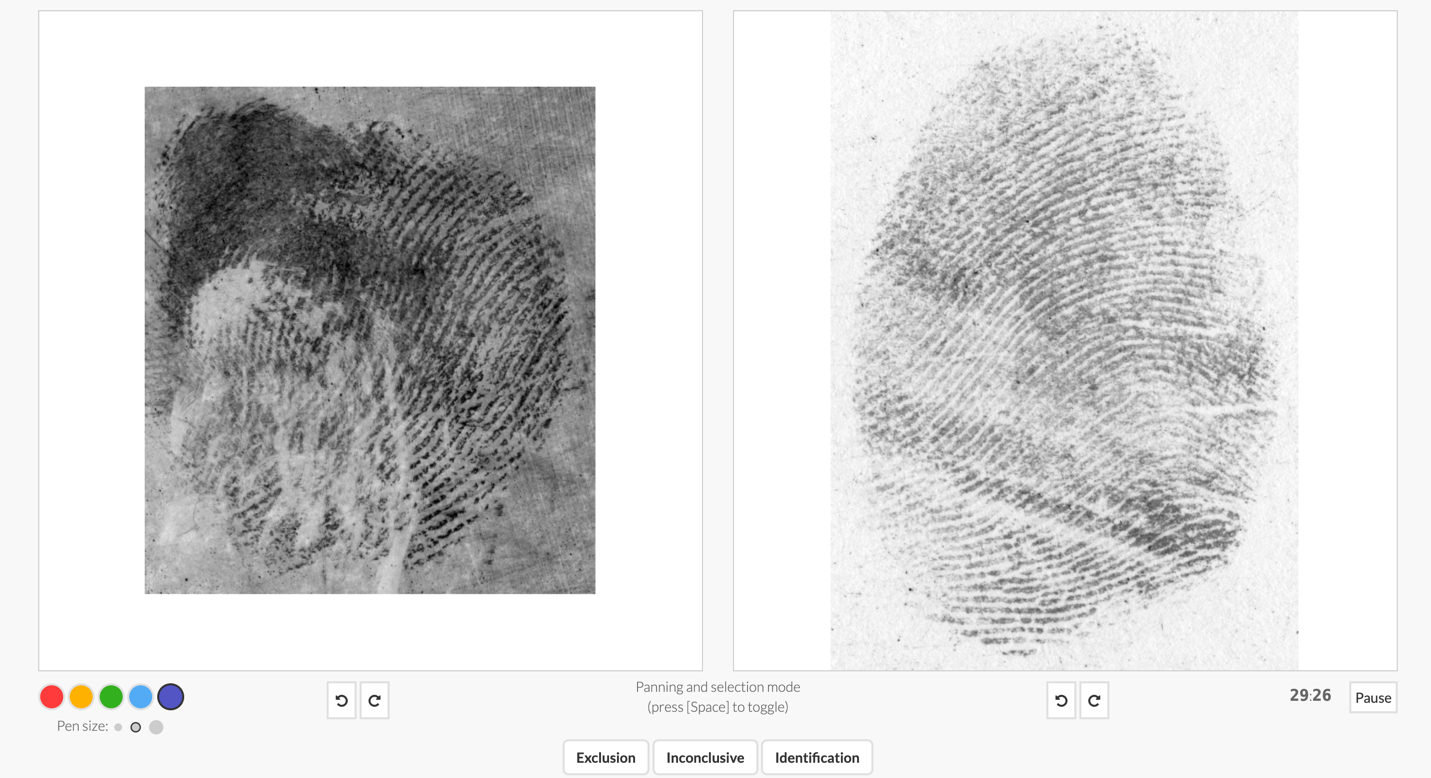


Fig S2: Example of a traditional comparison trial. First, examiners are presented with a latent print and asked to make a value determination using the following options: No value, Of Value for Exclusion, Of Value for Exclusion or Identification. Regardless of their decision, examiners then perform a comparison where they were presented with an exemplar print and asked to reach definitive conclusion using a three-conclusion scale (Identification/Inconclusive/Exclusion). Examiners were able to interact with the prints, including adding colored dots (marking up) impressions, zooming, and rotating images.
